# Computational technology for nasal cartilage-related clinical research and application

**DOI:** 10.1038/s41368-020-00089-y

**Published:** 2020-07-27

**Authors:** Bing Shi, Hanyao Huang

**Affiliations:** State Key Laboratory of Oral Diseases & National Clinical Research Center for Oral Diseases & Department of Oral Maxillofacial Surgery, West China Hospital of Stomatology, Sichuan University, 610041 Chengdu, China

**Keywords:** Dentistry, Oral anatomy, Medical research

## Abstract

Surgeons need to understand the effects of the nasal cartilage on facial morphology, the function of both soft tissues and hard tissues and nasal function when performing nasal surgery. In nasal cartilage-related surgery, the main goals for clinical research should include clarification of surgical goals, rationalization of surgical methods, precision and personalization of surgical design and preparation and improved convenience of doctor–patient communication. Computational technology has become an effective way to achieve these goals. Advances in three-dimensional (3D) imaging technology will promote nasal cartilage-related applications, including research on computational modelling technology, computational simulation technology, virtual surgery planning and 3D printing technology. These technologies are destined to revolutionize nasal surgery further. In this review, we summarize the advantages, latest findings and application progress of various computational technologies used in clinical nasal cartilage-related work and research. The application prospects of each technique are also discussed.

## Introduction

The nasal cartilage system contains two alar cartilages, the nasal septum cartilage, two upper lateral cartilages (also regarded as the extension of the nasal septum cartilage to both sides), and some accessory cartilage components. The nasal cartilage is considered the most demanding element of nasal surgery, especially of rhinoplasty and facial plastic surgery. The surgeon should understand the association of the nasal cartilage to the facial morphology, the function of both soft tissues and hard tissues, and nasal function. Patient-specific characteristics may also impact the outcomes of nasal surgery. Nasal surgery involving the nasal cartilage can be roughly divided into cosmetic surgery (no characteristic pathology or deformity of the nose; only performed to improve the appearance), deformity reconstruction (often related to developmental or trauma-related issues, such as cleft lip nose), functional reconstruction (such as repairing a nasal septum deviation or hypertrophy of the turbinate) and related operations such as tumour resection.

In recent years, biomedical engineering applications for the nasal cartilage has developed rapidly.^1,2^ Computational technology is one aspect of biomedical engineering that is used in clinical research, which forms the link between principle and practice. Computational technology can assist in exploring the role of physical factors and predict the results to guide the external conditions that should be applied in practice.^3^ The research on nasal cartilage-related surgery is conducted primarily to investigate the principles of surgical design and the changes in related physical factors (such as stress conditions, deformation or internal stress in the tissue) caused by the operation.^4–7^ It differs from basic research on nasal cartilage-related tissue engineering and should be verified by clinical operation and post-operative effect evaluation. Computational technology has become an important tool for nasal cartilage-related surgery and clinical application due to its high efficiency, low costs and ability to analyse and simulate organs and tissues independently.

The workflow for implementing computational technology is as follows: (1) apply imaging and model reconstruction technologies to build accurate models, (2) use structural mechanics and fluid mechanics analysis to explore biomechanical mechanisms and (3) use three-dimensional (3D) printing and computer-aided design (CAD) to assist clinical treatment. Using computational technology, we can target individual patient characteristics, such as the patient’s tissue anatomy and surgical requirements, and combine them with surgical methods to predict or obtain information that would be helpful for clinical treatment. Therefore, computational technology, mainly related to surgical modelling and simulation, can assist maxillofacial plastic surgeons in better understanding the nasal cartilage.^8–10^

**Fig. 1:**
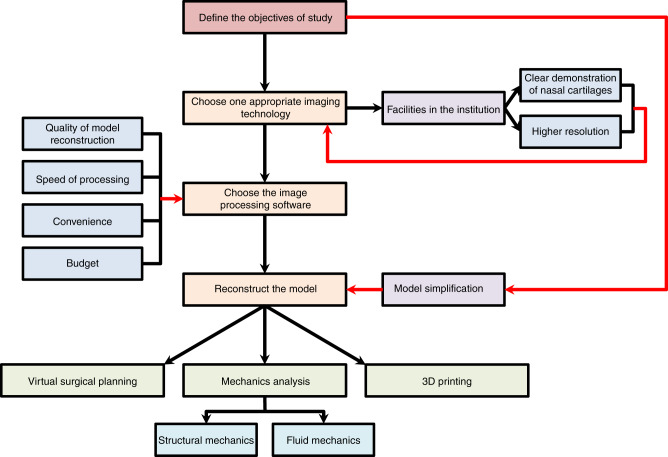
Flowchart for starting a nasal cartilage-related study based on computational technology. Arrows indicate the workflow of starting a nasal cartilage-related study based on computational technology. Black arrows indicate how to perform the study step by step; red arrows demonstrate how to make the decisions to choose the appropriate methods. The flowchart also demonstrates the structure of this review

Three-dimensional imaging technologies should form the basis for all patient-specific 3D model-related studies and applications. However, imaging of the nasal cartilages is more difficult than imaging of other cartilage types because of its small and complex structural features. Computational technology, such as structural and fluid mechanics analysis, can provide a reliable method to form the theoretical foundation for surgical technique modification. It can also directly help improve medical care, such as with virtual surgical planning and 3D printing, which have definitely increased surgical accuracy. In this review, we mainly introduce computational technologies related to the clinical research of the nasal cartilages, aiming to provide clinicians with alternative analytical methods, theoretical foundations and application techniques when selecting reasonable and effective treatment options for patients. A brief guideline demonstrating how this review is structured and the items to consider when starting a nasal cartilage-related clinical study using computational technology is shown in Fig. 1.

## 3D imaging and model reconstruction of the nasal cartilage

Three-dimensional imaging technology forms the basis of model reconstruction and is the foundation of the clinical patient-specific application of most computational technology. Precise and accurate 3D imaging of the nose structure is mandatory for completing a variety of subsequent computer processing steps. At present, one of the fundamental challenges in the preoperative evaluation of rhinoplasty is to distinguish the position and morphological characteristics of the nasal cartilage. Unlike articular cartilage, which is surrounded by synovial fluid, the nasal cartilage is surrounded by soft tissue, and articular cartilage is often larger than nasal cartilage.^11^ This discrepancy has led to limited planning and prediction of surgical outcomes, and it has also made it difficult to correlate the identified structures with functional outcomes.^12,13^ Conventional techniques currently used for 3D modelling of nasal cartilage include computerized tomography (CT) and magnetic resonance imaging (MRI). Since both methods of airway reconstruction can be applied, this section mainly discusses the 3D imaging methods used in the model reconstruction of nasal cartilage. Meanwhile, the 3D imaging of facial muscles is also discussed, as the facial muscles may also be included for a more complete nasal model.

### Computerized tomography

CT scans make use of computer-processed combinations of numerous X-ray measurements taken from different angles to produce cross-sectional images of specific areas of the human body, allowing the user to obtain tomographic images of the corresponding tissues. Cartilage is not easily visible on CT because of its similar X-ray attenuation to soft tissues, and it may often require the administration of contrast media for visualization. In contrast, CT is the best imaging method to evaluate bone due to its greater contrast resolution than soft tissues. CT can be applied to illustrate bony characteristics and quantify biologically relevant information, such as bone mineral density,^14^ which is calculated based on the concentration of calcium hydroxyapatite or dipotassium hydrogen phosphate in the bone.^15^ Contrast-enhanced CT is an expanded form of CT that utilizes iodinated contrast media (ICM) to evaluate the soft tissue; specifically, intra-articular injection is frequently suggested for knee evaluation in a technique named CT arthrography.^16^ ICM is used to contact the soft tissue structure. CT arthrography is regarded as the gold standard to evaluate the surface morphology of the articular surface, but it may not be applied to nasal cartilage. Administration of ICM through intravascular routes may help to reveal the structures in the nasal area, but this needs to be demonstrated in future studies, and the ability to show nasal cartilage has never been shown. However, ICM may suggest a clue for nasal-related muscle reconstruction. Quantitative CT arthrography can be used to identify the glycosaminoglycan (GAG) content,^17^ which may have the potential to distinguish nasal cartilage. ICM administration is also a problem for quantitative CT arthrography if applied to nasal cartilage. Cone beam CT (CBCT) has also been used in arthrography, and it shows good ability to separate the articular cartilage from the surrounding tissue.^18^ However, the characteristics of nasal cartilage, including its surrounding tissues, size and shape, make its visualization difficult on CBCT imaging, which limits the application of this technique to model reconstruction of the nasal cartilage.

**Fig. 2:**
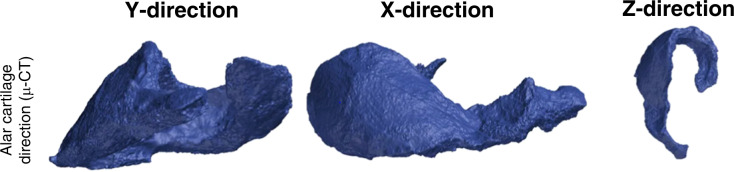
Nasal cartilage obtained from micro-CT scan. The three different views of the alar cartilage were obtained from micro-CT^20^

Unlike most other cartilage systems, the nasal cartilage system is small and complex and not surrounded by fluid but only soft tissue. Graviero et al.^19^ applied the method of stereorendering to CT scan results to show the morphological characteristics of nasal cartilage. Their study showed that this method could aid in improving the accuracy of the pathological diagnosis of the nose, but they did not verify that the reconstructed model could be applied to the nasal cartilage. The resolution of micro-CT is much higher than that of ordinary CT, with the cross-sectional pixel size reaching the micron level, which can improve the localization and morphological visualization of the nasal cartilage. Visscher et al.^20^ did not reconstruct the nasal cartilage structure with CT, but applied micro-CT to verify the effect of cartilage modelling (Fig. 2). Wu and Yin^21^ used micro-CT to scan nasal and lip tissue specimens from induced infants that were stained with 3.75% iodine-potassium iodide solution. Although primarily highlighting the muscle tissue, the general outline of nasal cartilage could also be seen.

A recent study compared the effects of micro-CT and clinically used CT on nasal cartilage imaging.^22^ In this study, Saxena et al.^22^ applied 60-μm resolution micro-CT and clinical CT under five different conditions to scan nasal tissue specimens and compared them with specimen sections to verify the scan results. The clinical protocols in this study were selected to span clinical care, maximize CT machine capabilities and compare CT-based visualization to the micro-CT scan. Clinical CT can show the characteristics of the nasal cartilage under specific conditions, but it is not as effective as micro-CT and cannot clearly distinguish the upper lateral cartilage from the alar cartilage. Because of the limited size of the scanning area, micro-CT cannot yet be used for facial scanning of normal people, but can only scan tissue samples, which will also limit its clinical application.

### Magnetic resonance imaging

MRI uses nuclear magnetic resonance to image body tissues. Compared with CT, MRI is more widely used for imaging soft tissues. MRI is also often applied to the detection and evaluation of cartilage in vivo. For MRI, the imaging sequences are important.^23^ Multiple different imaging sequences have been used and validated for articular cartilage evaluation. Delayed gadolinium-enhanced MRI of cartilage (dGEMRIC) can directly help to measure the proteoglycan/GAG content of cartilage and can also effectively evaluate cartilage regeneration, but this technique suffers from issues related to the toxicity of gadolinium administration and wait time.^24,25^ T1ρ can also be used to assess the proteoglycan/GAG content, but the evaluation is not as specific as dGEMRIC, as it also reflects other cartilage changes.^26^ In T2 mapping, increased T2 values can reflect the degeneration of cartilage, and this sequence can be read on most scanners.^27^ Decreased ultrashort echo time (UTE)‐T2* values can reflect cartilage matrix degeneration, while acute elevations in UTE‐T2* after anterior cruciate ligament injury may indicate irreversible cartilage damage and can help in evaluating the deep zone of cartilage.^28^ Sodium imaging is mostly associated with assessment of the GAG content, but its disadvantages include a low signal-to-noise ratio and the need for coils and high field strengths.^29^ Diffusion-weighted imaging can be used to evaluate the collagen structure, but the sequences are long and require high field strength similar to sodium imaging.^30^ In diffusion-tensor imaging, the mean diffusivity is correlated to proteoglycan concentration and anisotropy represents the collagen structure, but the disadvantages of diffusion-tensor imaging are the same as those of diffusion-weight imaging.^31^ GAG chemical exchange saturation transfer MRI can non-invasively quantify GAG content in cartilage without coils, but requires high field strength.^32^

For nasal cartilage, MRI has been widely used in the diagnosis of diseases and effect evaluation of treatments. Nasal cartilage tumours are often clinically imaged using MRI.^33,34^ The assessment of implanted tissue-engineered cartilage during rhinoplasty is also frequently examined by MRI.^35^ As early as 2001, Kleinheinz and Joosl^36^ used MRI to distinguish and evaluate cartilage and muscle tissue in patients with unilateral cleft lip and palate. The most significant advantage of MRI is that can be used to identify different soft tissues, and because of the various water and hydrocarbon contents of different kinds of tissue, MRI has also become the most potent tool for distinguishing cartilage.

**Fig. 3:**
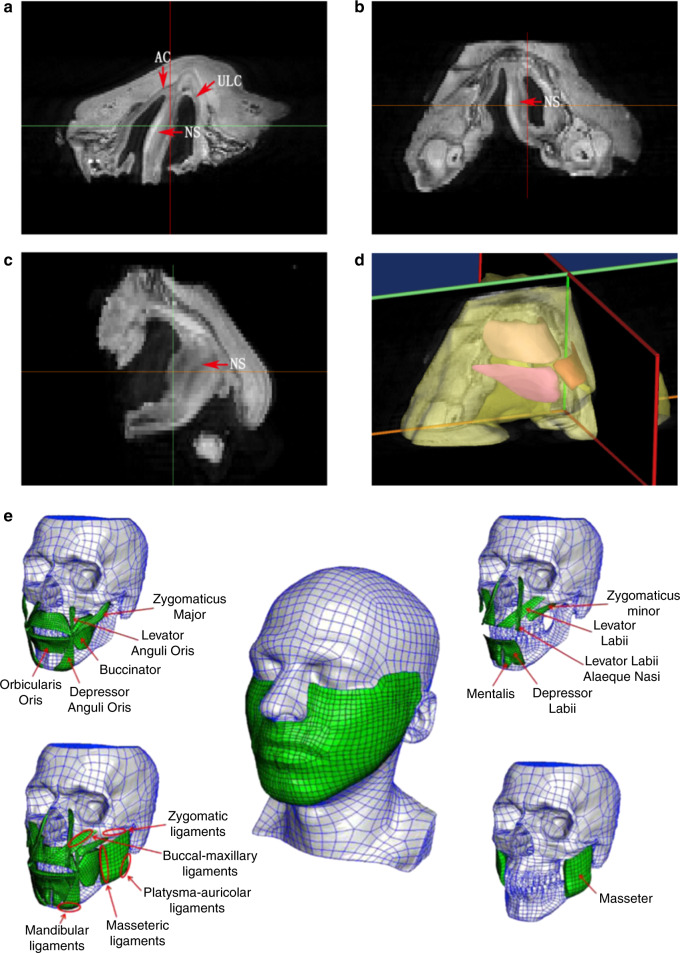
Nasal cartilage and facial muscles obtained from MRI scans. **a**–**c** Micro-MRI scan of the nasolabial region of an induced infant with unilateral complete cleft lip.^5,7^ The red arrow indicates the position of the cartilage. AC alar cartilage, ULC upper lateral cartilage, NS nasal septum cartilage. **d** 3D model reconstruction of the nasolabial region based on micro-MRI, including soft tissue and cartilage. **e** Facial muscles obtained from MRI^37^

MRI data of the nasolabial tissue have been used in nasal cartilage reconstruction. Its most significant advantage is that it can rebuild each patient-specific nasal cartilage structure based on individual patient data. Visscher et al.^20^ created a cartilage reconstruction model by scanning the nasal tissue of a corpse after fixation. Our team used MRI for the first time to scan the nasolabial part of healthy individuals and patients with unilateral cleft lip and nose deformities, reconstructed the cartilage structure of the nose and designed surgery based on the cartilage structure.^4,6^ Micro-MRI can also be used to scan smaller elements of the nasolabial tissues, resulting in higher resolution images. However, the very same small scanning range used in micro-CT also limits its scope for clinical application (Fig. 3a–d).^5,7^ More studies are required on applying different MRI imaging sequences to demonstrate the nasal cartilage and comparing these different methods. This would allow for better demonstrations of patient-specific nasal cartilage morphology.

Although cartilage is the most essential part of a nose model, the facial muscles that surround the nasal cartilage should also be included if a more accurate model is required. Like the nasal cartilage, the facial muscles are also small and thus share the same difficulties. The imaging segmentation for the different muscles is not as difficult as cartilage segmentation, however, because of the resolution of the imaging system, but it is still complicated, as there are many different muscles that surround the cartilage, such as the procerus, levator labii superioris alaeque nasi, compressor nasi, dilator nasi and depressor septi, and their directions and morphologies are all different. Especially for some small muscle bundles, it is difficult to determine the attachment site. Thus, simplification sometimes cannot be avoided, and researchers should pay much attention to how and what to simplify before they extract the imaging data for their study purposes. Mazza and Barbarino^37^ included many facial muscles in their study of a 3D mechanical model of facial soft tissue for surgery simulation. Those muscles associated with the nasal cartilage, including the levator labii alaeque nasi, levator labii and other facial functional muscles, were well reconstructed (Fig. 3e).

### Ultrasound imaging

Ultrasound imaging uses high-frequency sound waves to view inside the body. It has been applied to measure the nose and can show the shape of the nasal cartilage.^38,39^ It is difficult to reconstruct models based on 2D imaging, so 3D ultrasound imaging should be used for model reconstruction.^40^ Thus, 3D ultrasound imaging and related 3D reconstruction methods can also be very promising for scanning nasal cartilage, allowing for reductions in cost, elimination of radiation doses and maintaining the accuracy of the target tissues.

### Model reconstruction

With the continuous innovation of computational technology, the appropriate software for processing data visualization based on medical imaging can be chosen according to the needs and economic conditions of the researcher. Model reconstruction can be performed directly in the post-processing workstation that comes with most imaging equipment, or it can be finished with some commonly used medical image processing software, such as Mimics (Materialise, USA), OsiriX (Pixmeo, Swiss), Amira (Thermo Fisher Scientific, USA) and Avizo (Thermo Fisher Scientific, USA). Some domestic software, such as BioVision (BloomTek, China), ANYTHINK-GVCM (Crealife, China) and Trandomed (Trandomed, China), can also help handle imaging data nicely.

Commonly used medical image processing software can often complete most of the reconstruction work. If further modification or design of the model is required, software such as Geomagic (3D systems, USA) and 3D Max (Autodesk, USA) should be selected, and such software is also available for measurements.^4,41^ Multiple models can be assembled using CAD software, such as Creo Parametric (Parametric Technology Corporation, USA).^4,6^

**Fig. 4:**
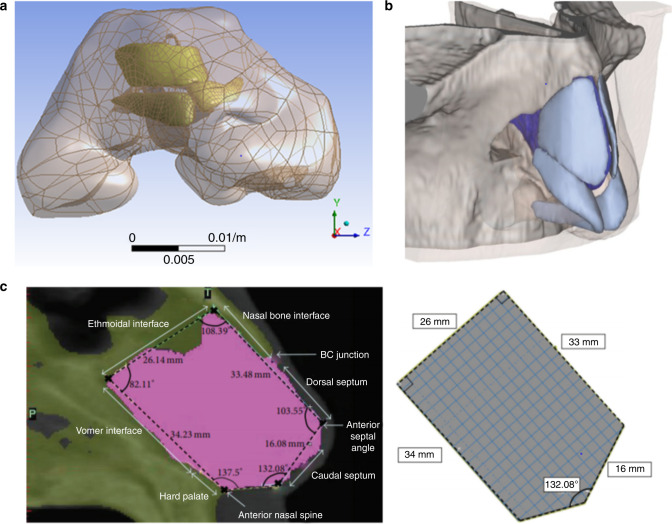
Different nasal cartilage reconstruction models used in different studies. **a** Patient-specific nasal cartilage reconstruction based on patient imaging data.^6^**b** Reconstructed soft tissue model based on patient imaging data and reconstructed cartilage based on soft tissue morphology.^42^**c** Simplified model of nasal cartilage^50^

Model reconstruction sometimes will be patient specific,^4,6^ in which the nasal cartilage model and nasal model or airway model reconstruction are based on patient image data. The process of individual model reconstruction is as follows: (1) DICOM format data are obtained by CT or MRI scans of the patient’s nasolabial region; (2) DICOM data are imported into the model reconstruction software for modelling, and anatomy-based segmentation is performed based on the threshold set on each slice. Because the area of nasal cartilage in each slice is small, manual segmentation is often required to obtain a more accurate model. The solid model is then exported in STL format; (3) computer-aided software is selected to design, modify and measure the reconstructed model if needed and to assemble the model for later processing (Fig. 4a).

Some theoretical studies do not require patient-specific models. Nasal cartilage models can be reconstructed based on experience and anatomy.^42^ The process for non-specific modelling is as follows: (1) DICOM format data are obtained by CT or MRI scans of the patient’s nasolabial region; (2) DICOM data are imported into the model reconstruction software for nasal soft tissue modelling, and the solid model is exported in STL format; (3) CAD software is used to reconstruct the nasal cartilage model based on the anatomical structure, and the output is exported in the STL format; and (4) the appropriate software is chosen to design, modify and measure the reconstructed model if needed and to assemble the model for later processing (Fig. 4b).

Some other theoretical studies only need to study the impact of the structure, so a more simplified nasal cartilage model is selected.^43^ In these cases, the software directly reconstructs the required structure in preparation for subsequent processing (Fig. 4c).

The above three model reconstruction cases are typically encountered in nasal cartilage research. For those models derived from the patient’s imaging data, brightness thresholding, region growing and manual segmentation are the keys to successful modelling. CT is good at showing hard tissues and automatic thresholding may help to save time, but when considering a small region such as that for a bone fracture, manual editing should be used. For MRI, deriving medium brightness tissue such as cartilage or demonstrating the connection between different soft tissues can take the bulk of time. During model reconstruction, the following should be kept in mind:^44^ (1) the models to be derived from the imaging; (2) the software to be used; and (3) the imaging segmentation tools to be used. This may help to find the right set of software and hardware for good model reconstruction.

## Computational simulation technology in clinical research of the nasal cartilage

Computational simulation is designed to predict the behaviour of a real-world or physical system-based mathematical model by computer. Computational simulations of tissues and organs can help researchers understand the influence of different and complex factors, such as engineering, anatomy, physics and mechanics, on a particular biomedical problem and assist in finding possible solutions. The most significant advantage of computational simulation technology is that it can ignore complicated influencing factors, observe the trend of activity of a single factor and provide directional guidance for clinical observation and treatment. Model simulation technology originated from the field of engineering. As mentioned in the introduction, the influence of physical factors on experimental results in tissue engineering can be guided by computational simulation. In clinical research, the quality of the surgical outcome often requires sufficient economic and time costs to be verified. It is not advisable to choose or modify the surgical method blindly. With the application of computational simulation technology, the surgical results can be predicted in advance to guide surgical practice, and the validity of the simulated results will be verified by the surgical outcomes, thereby further improving the therapeutic effects by forming a virtuous circle and reducing costs. Structural mechanics analysis and fluid mechanics analysis are the two most commonly used areas of computational simulation technology for research on the nose. Structural biomechanics analysis focuses on the effect of surgery on nasal morphology, while air dynamics analysis can be used to study the impact of surgery on nasal ventilation and other functions.

### Finite element method

The finite element method is the most commonly used method for both structural mechanics analysis and fluid mechanics analysis and requires a software to complete four steps: mesh development, preprocessing, solving and post processing.

Each step has a corresponding software or software module, and some software contains modules for all four steps, which can be selected according to the research requirements. Mimics (Materialise, USA), Ansys Workbench (ANSYS, USA)^42^ and FEBio (The University of Utah, USA)^45^ have been used for mesh generation and preprocessing in nasal cartilage and nasal-related research. Hypermesh (Altair Engineering, USA),^46^ Trelis (Csimsoft, USA)^47^ and CATIA (Dassault, USA)^48^ have been used for other kinds of cartilage in mesh generation and preprocessing and can also be applied to nasal cartilage. In addition to its convenient model reconstruction function, Mimics has certain advantages in surface 3D meshing. FEBio can be used for preprocessing, such as the calculation of superelastic coefficients and the extraction of non-linear characteristics. Abaqus (Dassault Systems, USA) has been widely used in finite element analysis,^49–51^ for example, in the analysis of porcine nasal cartilage by Chae et al.^52^ in 2001. Its advantage lies in structural mechanics analysis and performing non-linear calculations. Ansys Workbench contains a variety of analysis modules that are very convenient to use.^4–7^ COMSOL (COMSOL, USA), FEBio and SolidWorks (CASCIM, USA) have also been used for nasal cartilage-related finite element analysis.^41,42,45,53–56^

Finite element meshing is the most critical step for the finite element method. For complicated tissue or organ shapes, mesh generation is an extremely difficult and complicated process. Manual intervention for those structures is always needed.^57^ Different approaches can be chosen. Tetrahedral mesh generation is most commonly used in patient-specific applications, as it can be generated automatically. It can be easily used if the information of the model is available as a closed surface. Quality control of tetrahedral meshes is challenging for this automatic approach, however, and the Delaunay triangulation method,^58^ modified-octree technique,^59^ and advancing front technique^60^ can be applied to help in this process. The computation (CPU) time is related to the choice of mesh generator. For this approach, the main portion of the time will be spent extracting information from the patient’s images, such as for model reconstruction, rather than mesh generation, as it may only take a few minutes to generate the tetrahedral elements for a well-reconstructed model. Hexahedral and hexa-dominant mesh generation are other common approaches. Although it is a more accurate method, the manual generation of hexahedral elements takes up the bulk of time when performing patient-specific tissue modelling. Every single element requires the operator’s effort to complete. In patient-specific applications, there are no completely automatic approaches that are suitable for hexahedral meshing when targeting tissues or organs. Structured and unstructured grid generations are two types of meshing.^61,62^ Structured grid generation is based on rules for geometrical grid subdivisions and mapping techniques. For two-dimensional analyses, triangular or quadrilateral grids are applied, and hexahedral elements are used for three-dimensional methods. Unstructured grid generation is based on an explicit definition of the connections between nodes to form elements, in addition to the coordinates of the nodes themselves. Isogeometric analysis is another meshing method, which eliminates the finite element polynomial representation of geometry and replaces it with the representation.^63^ Meshless methods are also promising tools for surgical simulation, in which field variable interpolation is performed without the use of a predefined mesh.^64^ To summarize, there are a variety of meshing methods, and one should be chosen that can solve the research problem and meet the study requirements.

### Nasal cartilage-related structural mechanics analysis

Structural biomechanics analysis can be used to quantitatively study the deformations, stresses, energy and so on, produced by the 3D model of tissue structure under different influencing factors. Model reconstruction for nasal cartilage-related finite element analysis is performed according to the purpose of the study. The whole nose, including bone, cartilage and soft tissue, is not always required. When only targeting the mechanical properties of one aspect of the cartilage, such as the nasal septum or alar cartilage, only the model of this cartilage instead of that of the whole nose may be needed to perform computer-based mechanical testing. However, when targeting the influence of changes in the cartilage on the entire nose, the cartilage and soft tissue should be included in the model construction at a minimum because the nasal cartilage should be considered as one complete system. The different nasal cartilages are connected in the nasal system of humans, so changing one part will affect the others. Thus, the selection of the correct modelling strategy is the key to successful nasal finite element analysis.

The boundary conditions selected for the finite element analysis in structural biomechanics should be consistent with the actual research objectives. The constitutive equation can be set to be non-linear, linear, or an even more complicated system, but real human tissue cannot be simply defined. Appropriate simplification of the model is inevitable, and this is the reason why the boundary conditions of different studies are set differently.

The closer the computational simulation results are to reality, the richer the set environmental conditions and the more complicated the calculation process.^65^ At the same time, the setting of the physical properties of different tissues is also important. Taking nasal cartilage as an example, the physical and mechanical properties of different nasal cartilages or different parts from one cartilage are different.^56^ Important literature related to the finite element analysis of nasal cartilage is listed in Supplementary Table 1.

Simplifying the multi-factor, multi-condition and complex structure to analyse the influence of a single variable is still the most common method for nasal cartilage finite element analysis, and the simplification methods are also varied. At present, the nasal septum and the septal L-strut are the most frequently studied parts of the nasal anatomy using simplified models. Computers are used to design both the nasal septum model and the L-strut model to be as similar to reality as possible without involving other cartilages or soft tissues. By applying certain external factors (mostly different loading forces) or changing the side length or angle of the model, simulations of structural biomechanical changes under different conditions can be performed. Liong et al.^50^ obtained three types of deformation by loading forces on different parts of a simplified nasal septum model to correspond to nasal septum deflections observed in the clinic. Lee et al.^51^ explored the characteristics of stress distribution by changing conditions such as the angle and length of the nasal septum model and simulating the force on the nasal tip after trauma. A simplified nasal septum model was also applied in the analysis of the effect of laser treatment on the biomechanical properties of nasal cartilage.^66,67^

Simplified models of the septal L-strut have been studied many times. As early as 2007, Mau et al.^68^ combined the analysis of the force on the septal L-strut dissected from human specimens and the finite element analysis of the simplified L-strut in one study. Lee et al.^69^ analysed the deformation and stress distribution of the entire L-strut by changing the material properties and the support of the nasal tip and proposed the importance of maintaining adequate cartilage support at the bone–cartilage junction and nasal condyle. This study also compared its outcomes with those of Mau’s study. They further applied this model to analyse the minimum width and structural characteristics of the L-strut, which stabilizes the nasal septum, and indicated that only 1 cm was needed to support 45% of the whole width of the nasal septum.^43,70^

Simplified models of the alar cartilage are more complicated than those related to the nasal septum because the alar cartilage is a curved structure that supports the bilateral nasal alas and nasal columella. Oliaei et al.^54^ established three simplified models of the alar cartilage with different widths to simulate various cartilage resections and proposed maintaining at least 6 mm of lateral crus of the alar cartilage to ensure adequate structural support, for which sufficient width may be able to resist the force caused by post-operative scarring. The stress distribution in the tissue caused by the scar left after inverse V incisions on the columella was also analysed by a finite element model containing partial cartilage and soft tissue, which was set under non-linear hyperelastic conditions.^71^ Chang et al.^45^ recently measured the mechanical properties of nasal cartilage under non-linear conditions for the first time and verified the outcomes with the finite element method.

In 2014, Manuel et al.^42^ first included bone, cartilage and soft tissue in one finite element analysis to simulate the clinical problems encountered in functional rhinoplasty and pointed out that the nasal septum cartilage and most of the alar cartilage worked together to support the nasal tip. Based on Manuel’s model and research, Shamouelian et al.^72^ further investigated the two main mechanisms that support the nasal tip (the contact of the alar cartilage and the upper lateral cartilage and the contact of the medial crus of the alar cartilage and the nasal septum) and proposed that the second contact has a more significant effect on the nasal tip support. Leary et al.^55^ analysed the influence of different nasal cartilage resection widths on their strength and stability, using a model modification idea similar to that of Oliaei’s research.^54^ Tjoa et al.^73^ used Manuel’s model to simulate wound healing and surgical steps to explore their associations with inverted-V deformities. Gandy et al.^41^ analysed the effects of the size and shape of the columellar implant and its relationship with the medial crus of the alar cartilage on nasal tip support. From the series of studies by Manuel et al.,^42^ it was again shown that the same model can achieve the simulate different clinical problems through appropriate modification of the model and changes in the way that force is applied. At the same time, the model can also be used to analyse the function of each part of the structure. However, there are obvious problems in this series of studies. One is that the reconstruction of the nasal cartilage is not based on an individual patient’s image data, and the other is the lack of experimental or clinical data to further verify the results of the computer simulation.

**Fig. 5:**
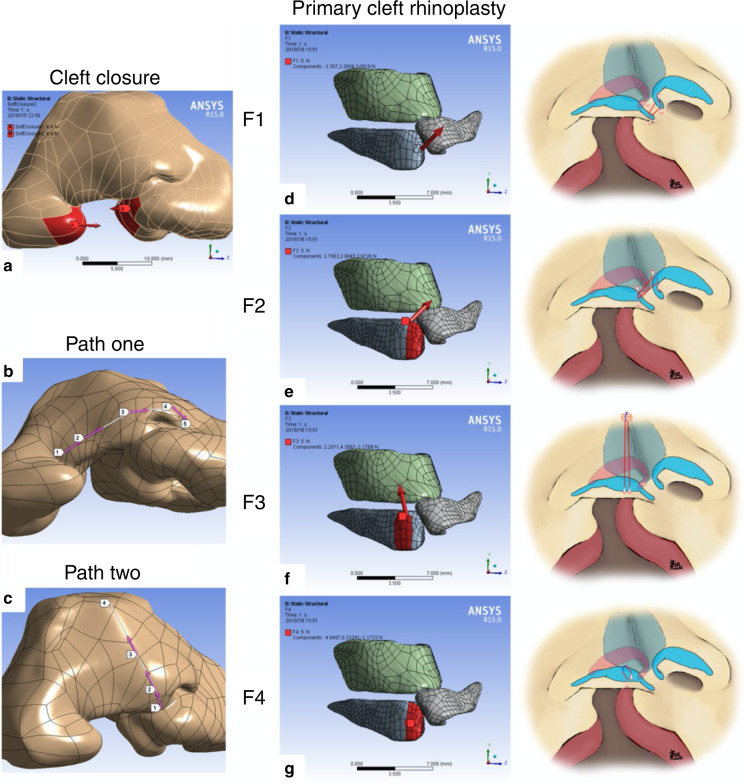
Finite element analyses of primary unilateral cleft lip rhinoplasty. **a** Two opposite forces at both sides of the cleft simulate the closure of a cleft lip. **b**, **c** Two paths illustrating the surface deformation and stress of the soft tissue. **d**–**g** The directions of force loading for the finite element simulation and schematic diagrams of four common suspension sutures during primary cleft lip rhinoplasty^6^

**Fig. 6:**
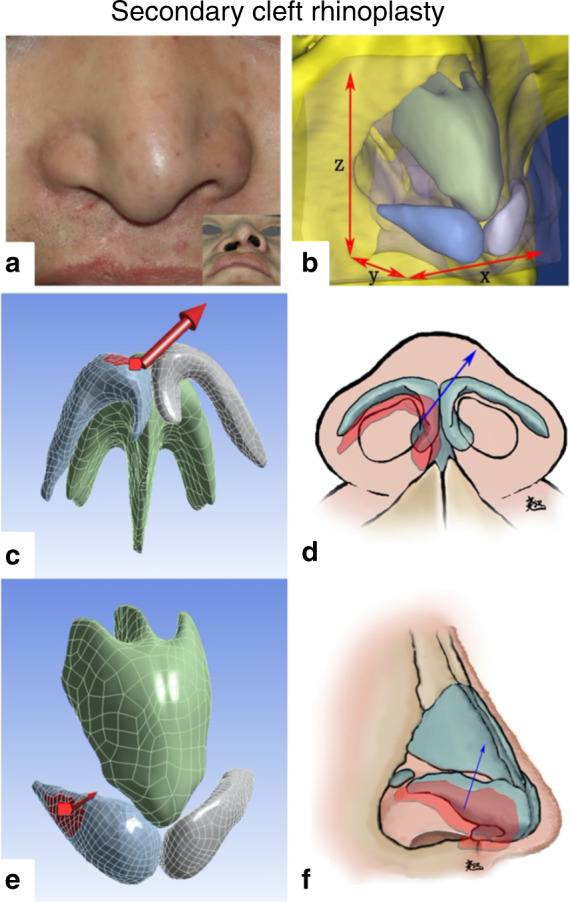
Finite element analyses of secondary unilateral cleft lip rhinoplasty. **a** Typical secondary unilateral cleft lip nasal deformity. **b** 3D reconstruction model of the patient-specific nose. **c**–**f** Schematic diagrams of two sutures in secondary cleft lip rhinoplasty and the directions of force loading during the finite element simulation^7^

Our team published a series of papers on the finite element analyses of the role of nasal cartilage in cleft lip rhinoplasty in 2018, and it was also the first study to reconstruct the nasal cartilage based on individual patient imaging data and combine the results with clinical outcomes. We first used finite element analysis to explore the pathological tethers on the nasal cartilage during the development of unilateral cleft lip nasal deformities and verified the main directions of these pathological tethers with clinical data.^4^ Next, we applied the finite element method to evaluate the effect of different alar cartilage suspension manoeuvres on a unilateral complete cleft lip nose model reconstructed from micro-MRI data and demonstrated the characteristics of different manoeuvres.^7^ Then, we explored the biomechanical forces that should be included during cleft lip rhinoplasty.^5^ We also used a secondary unilateral cleft lip nasal deformity model to simulate two suturing manoeuvres during unilateral cleft lip rhinoplasty, including the passive intercrural suture and a suture suspending the alar cartilage from the upper lateral cartilage. The functional biomechanical characteristics of the two sutures on the nasal structure were revealed, and clinical data were used to verify the finite element outcomes.^6^ This series of studies can be regarded as an example of “using finite element analysis to obtain a theoretical basis for guiding clinical practice” (Figs. 5, 6).

Most of the above studies assumed that the model was under linear elastic and homogeneous conditions. Although such an assumption can effectively achieve the purposes of the studies, ultimately, they are different from the conditions underlying the true, pathological situation and should affect the experimental results. The solution is therefore to emphasize the actual experimental or clinical results of the finite element outcomes and to complicate the simple conditions to simulate the real situation as much as possible. Implementing this solution is expected to be one of the directions for finite element studies for nasal cartilage analysis in the future. Meanwhile, finite element analysis of cartilage-related bioengineering scaffold materials has been widely used in the articular cartilage field, which is also expected to be a potential direction of nasal cartilage-related and even nasal chondrocyte-related research.^74,75^

A combination of finite element analysis and clinical practice should be specifically carried out in future studies. Finite element analysis should be regarded as an assistive tool for clinical research, as the verification of computational simulations is very important for reaching a conclusion. With simulations alone, discussing the association between the outcomes and the real situation will always be inconclusive, and the results will be limited to theory forever. When applying finite element analysis for surgery, it is recommended to perform the study as follows: (1) determine the clinical problems and design the modifications or solutions; (2) use computational simulations to predict the changes, compare the results to the objectives, and change the solution if the results do not meet the expectations; (3) practice the solutions to the clinical work and collect the data; and (4) compare the clinical outcomes with the computational results to finally decide if the problems have been solved. This kind of combination of theory and practice should help to impact this computer-simulating clinical research area.

### Nasal cartilage-related fluid mechanics analysis

The ventilation function of the upper airway is critical, and the first step of airflow into the upper airway is through the nasal airway. There are many methods for assessing ventilation function, including patient-reported quality of life questionnaires and physical examinations,^76^ and some measurement tools such as peak nasal rhinomanometry and acoustic rhinometry.^77,78^ Measurement of peak nasal inspiratory flow is also an effective method to assess nasal ventilation.^79^ Although these methods can help in determining the status of nasal ventilation function, they are unable to reveal the characteristics of nasal airflow and the causes of such airflows, such as local airflow and pressure changes and turbulence. Therefore, computational fluid dynamics (CFD) has become an effective method for analysing the characteristics of airway airflow. These computational techniques can not only quantify the various indexes of airflow in the airway, but can also be used to visualize the simulated airflow, which is impossible for other methods.^13,80–83^

CFD is a typical application of fluid mechanics, mathematics and computer science. Digital models are used to simulate the flow of liquids, particles and gasses in a flow field.^84^ Computational fluid mechanics need to follow appropriate calculation methods. The most basic algorithm is to apply Euler equations under inviscid fluid conditions and to apply Navier–Stokes equations under viscous fluid conditions. The workflow of nose-related CFD analysis is as follows: (1) reconstruct the airway model based on CT or MRI image data; (2) set boundary conditions (pressure, air velocity, etc.); and (3) perform fluid mechanics calculations and analysis.^85,86^ Typically, CFD is based on the principle of finite elements; that is, the entire airway needs to be meshed.^87^ ANSYS provides a series of modules based on finite element analysis, such as ICEM-CFD and Fluent, which can be used for fluid dynamic analyses of airways. However, the finite element method takes a very long time to complete just a single fluid mechanics analysis,^88^ and if the computer is not sufficiently configured, it will cause the computer to freeze or the operation to fail. The ANSYS Discovery Live processor module for the fluid mechanics analysis of velopharyngeal function recently published by our team can solve this problem well. The calculations performed by this software no longer require meshing the model.^89,90^ Regardless of the type of software used, much information can be obtained from the simulation, which is impossible for the other methods. Visualizing and quantifying airway fluid can significantly improve researchers’ and doctors’ understanding of the physiological functions of the nose.

Airway model reconstruction is much easier than nasal cartilage model reconstruction, as the airway has a clear boundary with the respiratory tract. For airways including the nasal cavity, nasopharynx, oropharynx, laryngopharynx and trachea, choosing the appropriate part of the airway is vital for CFD analyses. However, in aiming to analyse the airflow in the nose, one should not only reconstruct the nasal cavity, as the air in an entire flow field cannot be separated, and even a small change in the field could induce a change in the entire airflow pattern. For example, when the cross-sectional area of one large portion equals the total area of two small portions, the airflow patterns under the two conditions are totally different. Herein, researchers should choose the right parts of the airway based on their study design. The airway from the nostrils to the start of the trachea is recommended, as the morphology of the trachea and the distance to the nasal cavity should make the trachea have little influence on the airflow in the nasal cavity. Another aspect that should be considered is the collection of data from the sinuses, including the frontal sinus, ethmoid sinus, sphenoid sinus and maxillary sinus. The sinuses will definitely influence the airflow in the nasal cavity.^84^ For example, CFD showed that the magnitude and direction of the airflow pattern in an individual patient’s sinonasal anatomy after virtual endoscopic skull base surgery.^10,91^ However, the difficulty of sinus reconstruction is that it can only be observed if the sinus is connected to the nasal cavity after model reconstruction, and some small sinus models will cause the modelling process to be extremely complicated. The choice of whether to reconstruct the sinus should be based on the study purpose, but modelling should be as accurate as possible to simulate the real sinuses, so all the potential functional parts should be retained as best as possible.

The boundary conditions of the model need to be set before calculation. Common steps for setting the conditions for the nasal steady-state airflow field are as follows: (1) set a wall condition (zero velocity, stationary wall assumed) at the airway walls, (2) set a pressure-outlet condition with gauge pressure to 0^92^ and (3) set the speed or pressure-inlet condition associated with the research needs. This is because the analysis processes of different studies may involve inhalation or exhalation, and the corresponding states of different diseases are not consistent. When simulating the inhalation process, the inlet should be set at the nostrils, while for the exhalation process, the inlet should be set at the other side of the model. Changes to the velocity, pressure and inlets as well as the outlets over time in one simulation should also be applied when the goal of the study is to understand the airflow in the entire respiration process. At the same time, such changes will also cause the different settings of the equations, algorithms and physical properties of gases used in the simulation to change.^84,93^

**Fig. 7:**
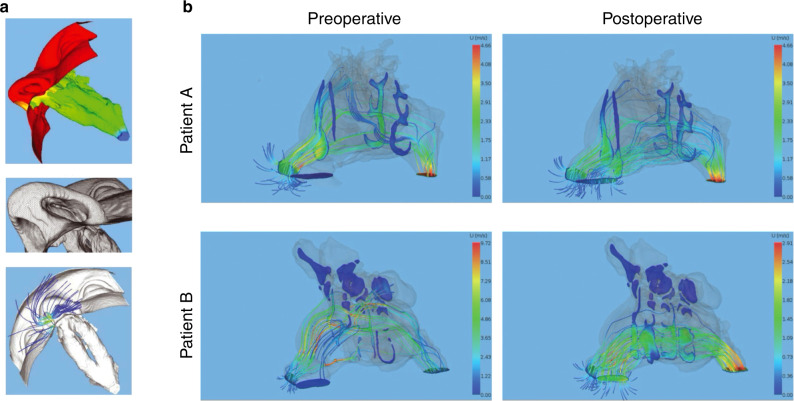
Computational fluid dynamics for handling nasal surgery. **a** CFD for analysing a patient-specific airway. **b** Streamlines and velocity contours show the post-surgical airflow changes for different patients. The changes in airflow through the nasal valve area can be clearly idenitified^101^

In 1995, Keyhani et al.^94^ established the first 3D model of the airway for dynamic airflow analysis, which was reconstructed based on CT data. Since then, CFD research has been widely employed in various studies related to the nose and airflow. In addition to the study of airflow, the use of CFD has also included changes in temperature and humidity^95–97^ and drug delivery through the respiratory tract.^98,99^ Of all the published CFD analyses of nasal airflow, the main direction has been to study nasal obstruction, the research of which mostly involves abnormal airway structure and surgical intervention (Fig. 7).^100–102^ However, studies that truly explore the effects of the nasal cartilage on airway airflow are not common. In such studies, the morphology of the airway should be given more attention than the shape of the cartilage, which would not change at the time of calculation. Therefore, when analysing fluid mechanics, it seems that how the nasal cartilage changes is not as crucial as the airway morphology supported by the nasal cartilage system. This perspective should be partly true. For instance, unilateral cleft lip nasal deformities will change the shape of the upper airway and then influence the airflow patterns. The fundamental reason for the deformity, however, is the displacement and deformation of the nasal cartilage complex.^8,9^

The nasal cavity after the nasal valve area is composed of bone, which acts as a stent, and is covered with soft tissue, while the nasal valve area is supported by the nasal cartilage system. Therefore, studies related to the nasal valve can be included in CFD analyses of the nasal cartilage. CFD showed that the nasal valve area accelerated the airflow owing to its resistance to airflow,^103,104^ and the pressure calculated by the computer was similar to the clinical pressure measurement.^105^ Changes in the cartilage, such as the collapse of the nasal cartilage and displacement of the nasal septum, can affect the nasal valve area,^106–109^ which would influence the patterns of airflow. Meanwhile, when a normal nasal airflow passes through the nostril and nasal valve area, the pattern of the airflow will also change as the nostril does, in which the nasal cartilage is similar to a scaffold.^110^

Although no study has directly incorporated cartilage in the airway 3D model and applied cartilage changes to simulate changes in airflow, some studies have compared the changes in airway airflow before and after surgery involving manipulation of the nasal cartilage. Shadfar et al.^111^ and Brandon et al.^112^ used fresh cadaver heads for nasal cartilage surgery, obtained a 3D model of the airway before and after the operation and performed CFD analyses. The first study proved that flare sutures could effectively improve airflow and add airflow resistance.^111^ The second study compared the airflow between spreader and butterfly grafts and suggested that the improvement in airflow from the perspective of CFD is similar.^112^ Virtually creating septal perforations with different sizes in different locations can also be regarded as manipulating the part of the nasal cartilage complex, and CFD showed alterations in nasal physiology following these structural changes.^113,114^

The above workflow provides an experimental procedure for analysing the effects of nasal cartilage changes on airway airflow, but it is worth mentioning that if the patient sample size is sufficient, researchers can choose to use individual patient image data before and after surgery for reconstruction and computational analysis. Furthermore, there is potential to modify the model in combination with structural mechanics analysis to simulate the surgical process and then perform CFD of the airway after the operation. The whole process is theoretical and can be compared with the real situation to obtain a more complete and logical research structure.

## Nasal cartilage-related virtual surgical planning and 3D printing

Unlike computational simulations, virtual surgical planning and 3D printing directly change clinical practice by making medical interventions truly patient specific. Virtual surgical planning changes traditional surgical designs, doctor–patient communications and medical education. 3D printing, meanwhile, allows linking the computational data to the real world. Both help to make the surgical process more accurate and improve medical quality.

### Virtual surgical planning

In the past few years, virtual surgical planning has gradually become an essential tool in the clinical work of craniofacial surgery.^115,116^ Virtual surgical planning, as well as CAD and computer-aided design manufacturing (CAM), is used in orthognathic, cranio-orbital and traumatic surgeries and microsurgeries involving craniofacial–facial bones. By acquiring the patient’s 3D image data, a software is used to reconstruct patient-specific tissues, and the surgical design can be performed on the reconstructed 3D model. Virtual surgical planning in orthognathic surgery is one ideal example.^117^ The advancements in 3D imaging techniques guarantee better visualization of any preoperative, intraoperative and post-operative phenotypic changes of both bone and soft tissues, which can help improve the accuracy of diagnosis and treatment planning. Furthermore, virtual planning allows fabrication of custom cutting guides and fixation plates, which can help surgeons reproduce virtual surgical planning osteotomies and save time for placement and removal of intraoperative splints. Orbital and traumatic reconstructions are also ideally suited for virtual planning, as CT allows a good view of the bony anatomy and critical neurovascular structures, which can help in efficiently planning osteotomies, fracture reductions and orbital implant placement.^118,119^

In the field of functional rhinoplasty, however, patient-specific virtual surgery planning has not been widely used, which may be due to the uniqueness of individual nasal structures. Appropriate digital modification of the patient’s computational models mimicking actual surgery and healing was proven to have the potential to predict post-operative outcomes and improve the success rates of surgery.^120,121^ Some reports applied patient-specific data to the design of rhinoplasty, but the analyses focused on the soft tissue without describing the nasal cartilage (one of the most important part of the nasal system), as CBCT or 3D photography cannot demonstrate clear cartilage information.^122–125^ In addition to only designing the external nasal shape, other studies used CFD to design the surgery, but these studies only extracted the patient’s airway model for analysis without involving the nasal cartilage.^126–128^ In virtual surgical planning for nasal airway obstruction, modification of the patient’s preoperative model was first performed, and CFD was applied to evaluate nasal patency.^120,121,129^ Zarrabi et al.^130^ reported the use of CAD/CAM to assist the reconstruction of the patient’s face. In this study, the designed scaffold was used to support a nasal prosthesis, which can be regarded as the prototype of the patient-specific design of the nasal cartilage.

However, medical education on rhinoplasty using computational 3D technology and digital simulation has been carried out since the beginning of this century. In 2002, Cutting et al.^131^ started to use 3D computational animations to demonstrate cleft lip and palate surgery for teaching. These animations describe the process of cleft lip rhinoplasty in detail, including the characteristics of the nasal cartilage at the site of the cleft lip nasal deformity and ways to manipulate the nasal cartilage during surgery. These animations described the methods for performing incisions and sewing the cartilage well and can aid medical students or surgeons understand the surgical process better. Different manoeuvres for cartilage suturing during cleft lip rhinoplasty were also shown along with an explanation. In recent years, the technology has been further developed, yielding digital simulations involved in human–computer interactions, which enabled students to study each surgical step in great detail.^132–134^ Each step of cleft lip nasal deformity reconstruction was listed. The students and doctors can choose the step they want to learn and view the manoeuvre from different angles. Videos of actual surgery were included and could be watched at the same time as the simulator was manipulated.

As we mentioned in the section on nasal cartilage imaging, our team first performed MRI scans of patients to obtain patient-specific nasal cartilage information in 2018. As virtual surgery planning requires patient-specific data, the application of virtual surgery planning to nasal cartilage was delayed with respect to that for bone and soft tissue. Computational imaging technology further limits this application. There are two main problems with nasal cartilage reconstruction: (1) the resolution of ordinary MRI is low, and micro-MRI cannot be used for patient scanning; and (2) as a result of the poor image resolution, it always takes much time to perform artificial reconstruction of the nasal cartilage, which also limits its clinical application. However, it is believed that as the technology continues to be developed, these problems will be gradually solved, and virtual surgery planning will become well applied to nasal cartilage-related surgery.

### 3D printing

3D printing is a manufacturing technique that can be used to build complex 3D geometric structures, which can be used as scaffolds for tissue engineering or patient-specific implantation. 3D printing technology was first developed in 1990 and after approximately a decade, was implemented in the medical field to perform bioprinting.^118^ The function of 3D printing in current clinical applications is mostly to build patient-specific implants following the principles of precision medicine.^135^ In rhinoplasty, cartilage implantation is widely used, and the current sources of implanted bone are mostly from the patient’s auricle or rib cartilage.^136,137^ Commercial implants can also be chosen.^138,139^ The creation of appropriate implants is not only limited by the quality of the cartilage, but can also cause secondary trauma to the patient. 3D printing has now become the method of choice for the accurate design and construction of patient-specific implants.^140–142^ Tissue engineering cartilage is expected to be a solution to the above problem, and 3D bioprinting will become the leading force in the research of tissue engineering cartilage implants. 3D bioprinting is the construction of active tissues or organs in vitro based on the principles of 3D printing.^143^ The development of bioinks, which are biocompatible and can contain other biological components such as cells or bioactive factors, was essential in promoting the application of 3D printing to tissue engineering.^118^ Herein, 3D printing for the nasal cartilage area could encompass two kinds of targets: (1) based on tissue engineering, 3D bioprinting could be used to fabricate a scaffold for regenerating the biological cartilage, which would then be applied or implanted to the defect area; or (2) 3D printing could be used directly to build a biocompatible scaffold or implant to function as the cartilage itself or to fabricate the prosthesis, in which biological cartilage is not needed.

Inkjet, laser assisted and extrusion are the three main 3D printing methodologies.^144^ The inkjet method creates a pressure change upstream of the nozzles to eject the material droplet. High-speed printability, low costs and the possibility of encapsulating cells in the material are the advantages of the inkjet method. The laser-assisted method does not require nozzles. During printing, a laser pulse is used to stimulate the target area, and the energy-absorbing layer is evaporated, leading to the formation of a droplet. The extrusion method is the most commonly used method, in which the material fuses to create a continuous structure after leaving the nozzles at room temperature. Affordability, high speed and the ability to print multiple materials at the same time with a multi-nozzle printer are the advantages of the extrusion method. 3D printing materials for tissue engineering can be summarized into four types: polymers, ceramics, composites and cell aggregates.

For the first nasal cartilage target, with the development of tissue engineering, four elements have gradually been developed: cells, scaffolds, bioactive factors and physical features.^145^ Different cells,^146–148^ scaffolds from different materials,^149–153^ and different bioactive factors^154,155^ have been applied to the tissue engineering of nasal cartilage and have achieved good results, but tissue engineering related to physical factors is more common in other types of cartilage.^156,157^ Nevertheless, the 3D bioprinting of nasal cartilage remains in the experimental stage because it is limited by the clinical application of related bioactive factors. This method has the potential to improve cartilage engineering.^158–160^

**Fig. 8:**
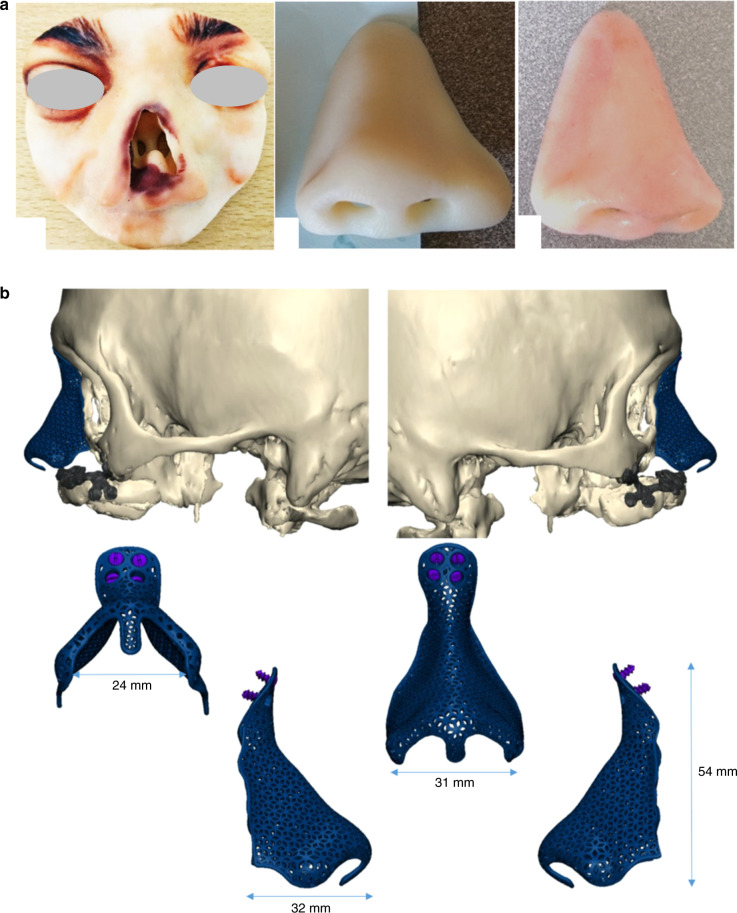
Application of 3D printing in nasal cartilage-related surgery. **a** 3D printing of a patient-specific nasal prosthesis.^162^**b** Patient-specific design and 3D printing of a meshed titanium nasal prosthesis^165^

Therefore, the second target is what we can currently achieve apply to clinical work. In recent years, the 3D printing process for nasal prostheses is as follows: (1) complete the patient-specific design on the computer; (2) select the appropriate materials for 3D printing; and (3) print the patient-specific nasal prosthesis (Fig. 8a).^161,162^ The 3D-printed nasal implant or stent can be regarded as a substitute for the nasal cartilage and has been gradually applied to rhinoplasty. In nasal septum perforation, 3D printing has been used to make a prosthesis that fits the size of the perforation and acts as the nasal septum cartilage.^163^ In nasal septum deviation, a stent to correct the deviation has also been accurately manufactured by 3D printing.^164^ A 3D-printed, porous titanium scaffold can also act as the nasal cartilage and was combined with a skin flap for nasal reconstruction (Fig. 8b).^165^ The implants used in rhinoplasty can be individually designed according to the needs of the patient, and the required shape and size of the implants can be calculated. The implants can be accurately manufactured by 3D printing and finally applied during surgery, and the surgical outcomes are acceptable.^166,167^ The addition of cells to the implants for tissue engineering according to the concept of 3D bioprinting will promote the application of 3D printing in rhinoplasty in the future.^168^

## Conclusions

Computational technology has great potential for clinical application and research in the field of nasal cartilages. The rational use of these methods can lead to the clarification of surgical goals, rationalization of surgical approaches, increased precision and personalization of surgical design and preparation, and improved convenience of doctor–patient communication. Meanwhile, the application of computational technology will also reduce medical costs and improve medical efficiency. Advances in 3D imaging technology will promote nasal cartilage-related applications and research, including computational modelling technology, computational simulation technology, virtual surgery planning and 3D printing technology, which are destined to revolutionize nasal surgery further.

## Supplementary information

Supplementary information
