# Supplementary material for: Computational technology for nasal cartilage-related clinical research and application
Source: Int J Oral Sci. 2020 Jul 27;12:21. doi: 10.1038/s41368-020-00089-y (PMC7385163; doi:10.1038/s41368-020-00089-y)
Supplement: Supplementary file 1 — Supplementary information [file 41368_2020_89_MOESM1_ESM.docx]

#### Supplementary Information

**Supplementary Table 1 Important literatures related to finite element analysis of nasal cartilage**

| References | Time | Purpose of Finite Element Analysis | Model Composition | Model Reconstruction | Elastic modulus (*E*, MPa), Poisson's ratio (*v*) and density (*ρ*，Kg/m^3^) | Constitutive equation |
| --- | --- | --- | --- | --- | --- | --- |
| Protsenko et al. ^66,67^ | 2007 | Changes of laser-assisted straightening of the septum | Septum simplified model | Modeling from measurement data | *E*: 6.1 and 0.7; *v*: 0.2 and 0.3 | Homogeneous, linear elastic model |
| Mau et al. ^68^ | 2007 | Stability of different L-struts | L-strut simplified model | Modeling from measurement data | NA | Homogeneous, linear elastic model |
| Lee et al. ^69^ | 2010 | Mechanical differences of different L-struts | L-strut simplified model | Modeling from measurement data | *E*: 0.44 and 5.0; *v*: 0.32 and 0.38 | Homogeneous, linear elastic model |
| Lee et al. ^51^ | 2010 | Changes of septum after trauma | Septum simplified model | Modeling from measurement data | NA | Homogeneous, linear elastic model |
| Gizzi et al. ^71^ | 2012 | The scar’s function on the columella | Soft tissue and cartilage | Modeling from measurement data | NA | Heterogeneous, nonlinear hyperelastic model |
| Oliaei et al. ^54^ | 2012 | Validation of experimental data from nasal cartilage test | Alar cartilage simplified model | Modeling from measurement data | *E*: 5.0; *v*: 0.33 | NA |
| Liong et al. ^50^ | 2013 | Deformation of septum under different forces | Septum simplified model | Modeling from measurement data | *E*: 5.0; *v*: 0.33; *ρ*: 2000 | Homogeneous, linear elastic model |
| Manuel et al. ^42^ | 2014 | The relation between nasal cartilages and nasal tip projection under tip collapse situation | Bone, cartilage and soft tissue | Bone and soft tissue remodeled based on CT; Cartilage remodeled based on anatomy and experience | (1) Bone: *E*: 15000; *v*: 0.22; *ρ*: 1900; (2) Cartilage: *E*: 0.8; *v*: 0.15; *ρ*: 1080; (3) Soft tissue: *E*: 0.5; *v*: 0.33; *ρ*: 980 | Homogeneous, linear elastic model |
| Shamouelian et al. ^72^ | 2015 | The nasal cartilages’ influence on nasal tip projection | Bone, cartilage and soft tissue | Bone and soft tissue remodeled based on CT; Cartilage remodeled based on anatomy and experience | (1) Bone: *E*: 15000; *v*: 0.22; *ρ*: 1900; (2) Cartilage: *E*: 0.8; *v*: 0.15; *ρ*: 1080; (3) Soft tissue: *E*: 0.5; *v*: 0.33; *ρ*: 980 | Homogeneous, linear elastic model |
| Lee et al. ^70^ | 2015 | The surgery’s influence on L-strut | L-strut simplified model | Modeling from measurement data | *E*: 0.41; *v*: 0.30 | Homogeneous, linear elastic model |
| Leary et al. ^55^ | 2015 | The alar cartilages’ effect on stability of nasal tip | Bone, cartilage and soft tissue | Bone and soft tissue remodeled based on CT; Cartilage remodeled based on anatomy and experience | (1) Bone: *E*: 15000; *v*: 0.22; *ρ*: 1900; (2) Cartilage: *E*: 0.8; *v*: 0.15; *ρ*: 1080; (3) Soft tissue: *E*: 0.5; *v*: 0.33; *ρ*: 980 | Homogeneous, linear elastic model |
| Tjoa et al. ^73^ | 2016 | The relation between upper lateral cartilages and inverted-V deformity | Bone, cartilage and soft tissue | Bone and soft tissue remodeled based on CT; Cartilage remodeled based on anatomy and experience | (1) Bone: *E*: 15000; *v*: 0.22; *ρ*: 1900; (2) Cartilage: *E*: 0.8; *v*: 0.15; *ρ*: 1080; (3) Soft tissue: *E*: 0.5; *v*: 0.33; *ρ*: 980 | Homogeneous, linear elastic model |
| Gandy et al. ^41^ | 2016 | Columella implants’ influence on nasal tip | Bone, cartilage and soft tissue | Bone and soft tissue remodeled based on CT; Cartilage remodeled based on anatomy and experience | (1) Bone: *E*: 15000; *v*: 0.22; *ρ*: 1900; (2) Cartilage: *E*: 0.8; *v*: 0.15; *ρ*: 1080; (3) Soft tissue: *E*: 0.5; *v*: 0.33; *ρ*: 980 | Homogeneous, linear elastic model |
| Lee et al. ^43^ | 2016 | The relation between different L-struts and collapse | L-strut simplified model | Modeling from measurement data | *E*: 0.41; *v*: 0.30 | Homogeneous, linear elastic model |
| Huang et al. ^4^ | 2018 | The pathological forces on cleft lip nasal cartilages | Cartilage and soft tissue | Modeling based on MRI | (1) Cartilage: *E*: 0.8; *v*: 0.15; *ρ*: 1080; (2) Soft tissue: *E*: 0.5; *v*: 0.33; *ρ*: 980 | Homogeneous, linear elastic model |
| Huang et al. ^6^ | 2018 | Cartilage changes during secondary cleft lip rhinoplasty | Cartilage and soft tissue | Modeling based on MRI | (1) Cartilage: *E*: 0.8; *v*: 0.15; *ρ*: 1080; (2) Soft tissue: *E*: 0.5; *v*: 0.33; *ρ*: 980 | Homogeneous, linear elastic model |
| Huang et al. ^5,7^ | 2018, 2019 | Cartilage changes during primary cleft lip rhinoplasty | Cartilage and soft tissue | Modeling based on Micro-MRI | (1) Cartilage: *E*: 0.8; *v*: 0.15; *ρ*: 1080; (2) Soft tissue: *E*: 0.5; *v*: 0.33; *ρ*: 980 | Homogeneous, linear elastic model |
| Chang et al. ^45^ | 2019 | Measurement of the mechanical properties of the nasal cartilages | Cartilage | Modeling based on specimen | NA | Nonlinear elastic model |

**Note: NA refers to relevant data not mentioned in the article.**
